# Supplementary material for: Loss of TET function in T regulatory cells yields ex-Treg cells biased toward T follicular helper cells, causing autoimmune diseases through autoantibody production
Source: bioRxiv. 2025 Sep 3:2025.08.29.673187. Preprint. [Version 1] doi: 10.1101/2025.08.29.673187 (PMC12424951; doi:10.1101/2025.08.29.673187)

bioRxiv preprint doi: https://doi.org/10.1101/2025.08.29.673187; this version posted September 3, 2025. The copyright holder for this preprint (which was not certified by peer review) is the author/funder, who has granted bioRxiv a license to display the preprint in perpetuity. It is made available under aCC-BY 4.0 International license.

Suppl. Figure 1

A-C. RNA-seq analysis for $CD4^+$ YFP($FOXP3^-$) T cells from *Foxp3Cre WT* mice and *Foxp3-Cre Tet2/3^{fl/fl}* mice (14-weeks-old). **A.** PCA plot. **B-C.** Mean average (MA) plot of genes differentially expressed in DKO-moderate relative to their expression in WT (**B**), DKO-severe relative to their expression in WT (**C**). **D.** The graphs show the *Tet2* expression in *Tet2/3 DKO* Treg cells relative to WT Treg cells (*left*), the *Tet2* expression in *Tet2/3 DKO* not-Treg cells relative to WT not Treg cells (*right*). Treg cells: $CD4^+$ YFP(FOXP3)$^+$, WT not Treg cells: $CD4^+$ YFP(FOXP3)$^-$, Tfh cells: $CD4^+$ YFP(FOXP3)$^-$ PD-1$^+$ $CXCR5^+$, not Tfh cells: $CD4^+$ YFP(FOXP3)$^-$ PD-1$^-$ $CXCR5^-$, Naïve $CD4^+$ cells: $CD4^+$ YFP(FOXP3)$^-$ $CD62L^{high}$ $CD44^{low}$.

Suppl. Figure 2

A-C. Flow cytometry analysis of cytokines in $CD4^+$ and $CD8^+$ cells. IL-17A$^+$ cells and IFN-γ$^+$ cells with PMA and ionomycin stimulation (gated on TCRβ$^+$ $CD4^+$ cells) (**A**), IL-17A$^+$ and IFN-γ$^+$ cells with PMA and ionomycin stimulation (gated on TCRβ$^+$ $CD8^+$ cells) (**B**), Perforin$^+$ and Granzyme B$^+$ cells (gated on TCRβ$^+$ $CD8^+$ cells) (**C**) in pooled spleen and peripheral lymph nodes (cervical and inguinal) of from 14-weeks-old *Foxp3Cre WT* and *Foxp3-Cre Tet2/3^{fl/fl}* mice. **D-E.** Quantification of the frequency of IL-17A$^+$ cells (**D**), IFN-γ$^+$ cells (**E**) in $CD4^+$ T cells. **F-H.** IFN-γ$^+$ (**F**), Perforin$^+$ cells (**G**), Granzyme B$^+$ cells (**H**) in $CD8^+$ T cells. **I.** Quantification of the frequency of $B220^+$ cells in live cells and absolute number. **J.** Flow cytometry analysis of GC-B cells (Fas$^+$ GL-7$^+$ cells) (gated on $B220^+$ cells) in pooled spleen and peripheral LNs (cervical and inguinal) from 14-weeks-old *Foxp3Cre WT* and *Foxp3-Cre Tet2/3^{fl/fl}* mice (*left*). Quantification of the frequency of Fas$^+$ GL-7$^+$ cells in $B220^+$ T cells.

Suppl. Figure 3

A. The UMAP plot showed 0-19 clusters from sc-RNA sequencing. Annotated clusters are shown. TCRβ$^+$ cells were sorted from pooled spleen and peripheral lymph nodes (cervical and inguinal) from *Foxp3Cre WT* mice and *DKO-severe Foxp3-Cre Tet2/3^{fl/fl}* mice (14-weeks-old) and sc-RNA sequencing was performed (n=1).

Suppl. Figure 4

A. Heatmap from polyreactivity assay for serum isolated from WT (n=8) and DKO-moderate (n=3), DKO-severe (n=4)

bioRxiv preprint doi: https://doi.org/10.1101/2025.08.29.673187; this version posted September 3, 2025. The copyright holder for this preprint (which was not certified by peer review) is the author/funder, who has granted bioRxiv a license to display the preprint in perpetuity. It is made available under aCC-BY 4.0 International license.

tested by autoantibody array assay kit (13-15 weeks old mice). For data analysis, R package ‘limma’ and multiple comparisons correction was performed (adjusted p value < 0.05 between tested groups).

Suppl. Figure 5

A. H&E staining of Kidney, Liver, Lung and Pancreas from 14-week-old *Foxp3Cre WT* and *Foxp3-Cre Tet2/3$^{fl/fl}$* mice. Scale bar; Kidney: 100μm, Liver: 200μm, Lung: 250μm, Pancreas: 50μm.

Suppl. Figure 6

A-C. Genome browser views showing 5mC% (Track 1), 5hmC% (Track 2) from 6 base sequencing, gene expression (RNA-seq, track 3) in *Tox2* (**A**), *Maf* (**B**) and *Batf* (**C**) locus. 6 base sequencing; WT: naïve CD4$^+$ T cells (CD4$^+$ YFP(FOXP3)$^-$ CD62L^{high} CD44^{low}) from *Foxp3Cre WT* mice, DKO: Tfh like cells (CD4$^+$ YFP(FOXP3)$^-$ PD-1$^+$ CXCR5$^+$) from DKO-severe *Foxp3-Cre Tet2/3$^{fl/fl}$* mice. RNA seq; WT: CD4$^+$ YFP(FOXP3)$^-$ T cells from *Foxp3Cre WT* mice, DKO: CD4$^+$ YFP(FOXP3)$^-$ T cells from DKO-severe *Foxp3-Cre Tet2/3$^{fl/fl}$* mice.

bioRxiv preprint doi: https://doi.org/10.1101/2025.08.29.673187; this version posted September 3, 2025. The copyright holder for this preprint (which was not certified by peer review) is the author/funder, who has granted bioRxiv a license to display the preprint in perpetuity. It is made available under aCC-BY 4.0 International license.

Suppl Figure 1

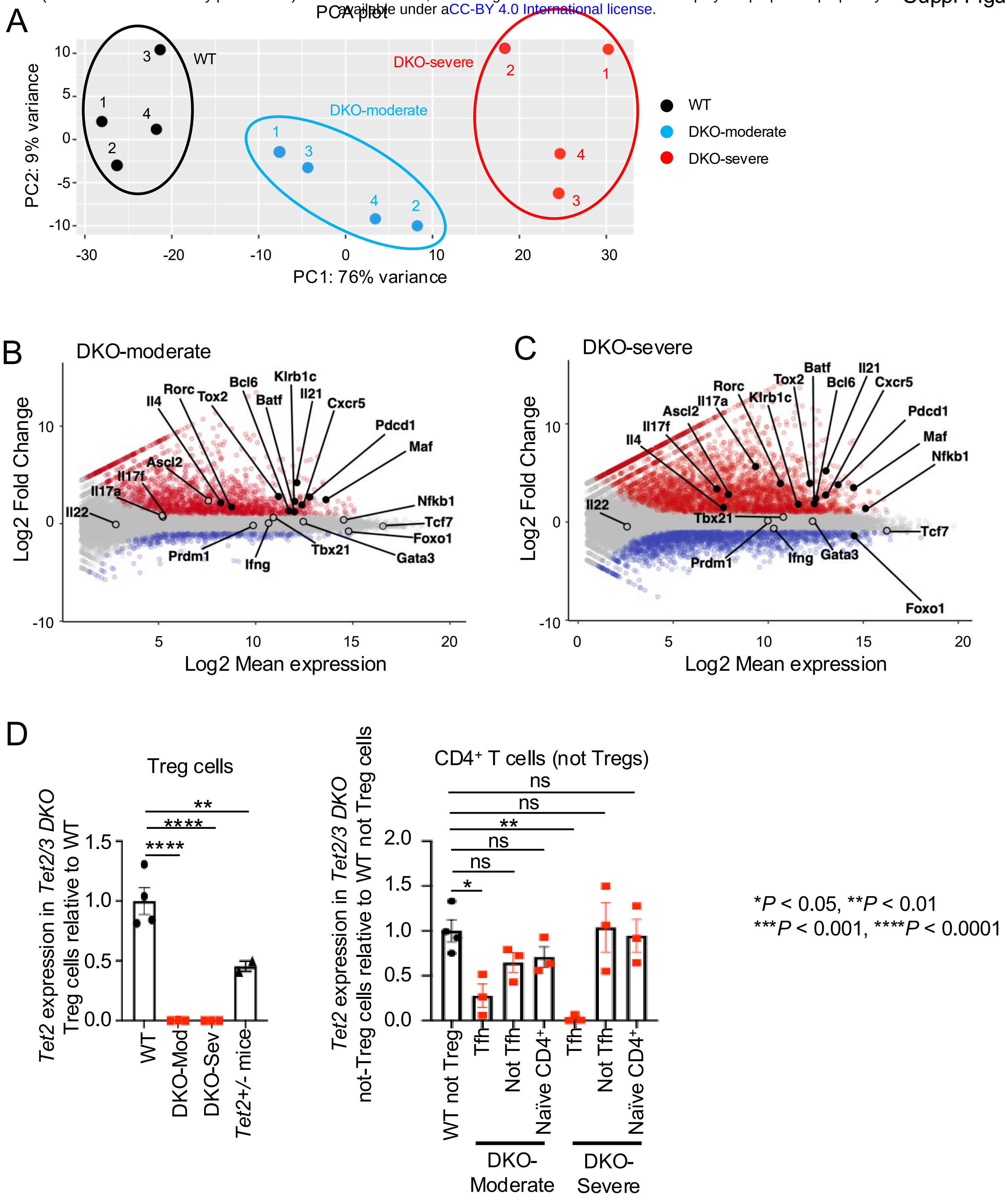

bioRxiv preprint doi: https://doi.org/10.1101/2025.08.29.673187; this version posted September 3, 2025. The copyright holder for this preprint (which was not certified by peer review) is the author/funder, who has granted bioRxiv a license to display the preprint in perpetuity. It is made available under aCC-BY 4.0 International license.

Suppl Figure 2

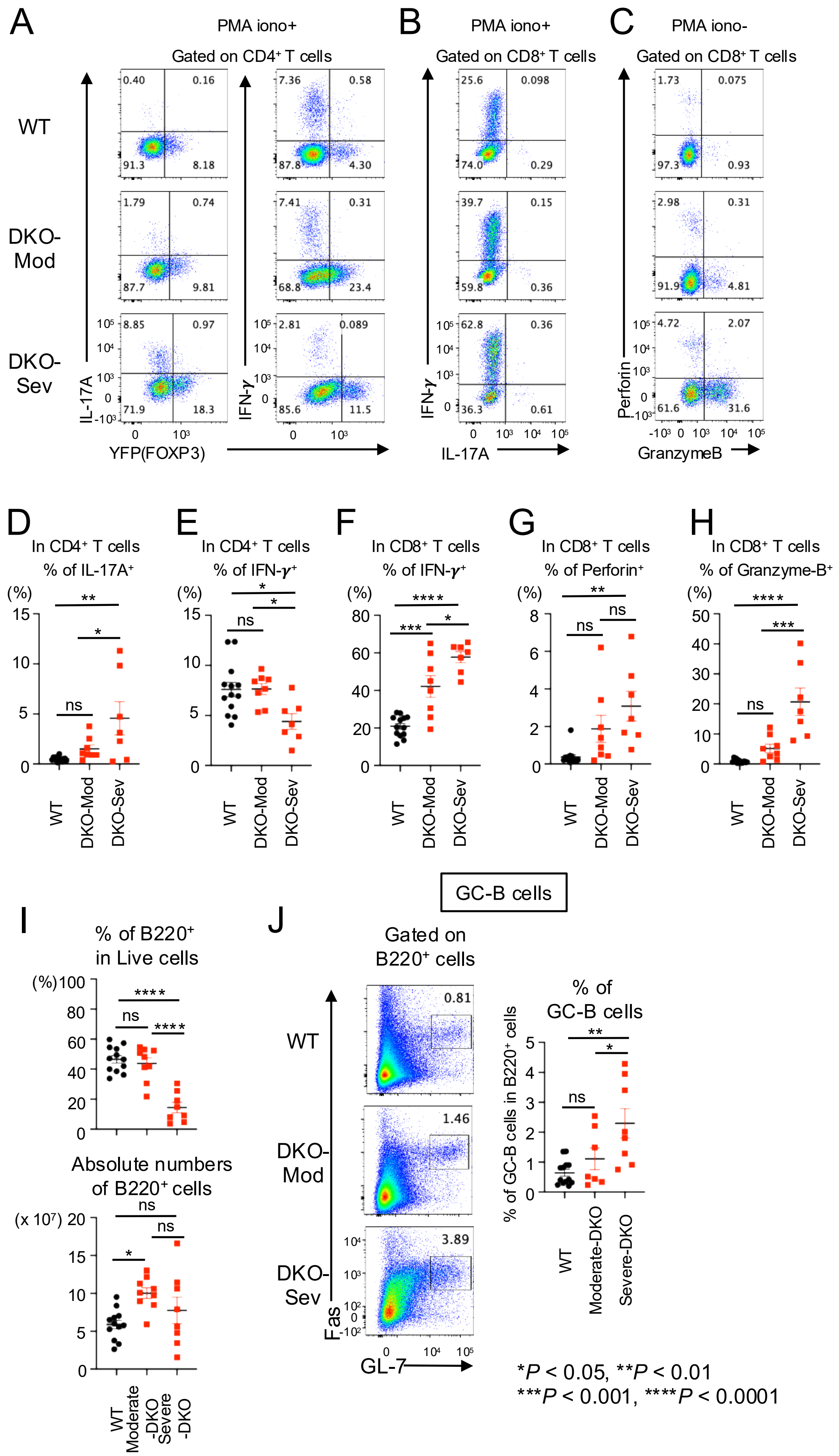

*$P < 0.05$, **$P < 0.01$
***$P < 0.001$, ****$P < 0.0001$

bioRxiv preprint doi: https://doi.org/10.1101/2025.08.29.673187; this version posted September 3, 2025. The copyright holder for this preprint (which was not certified by peer review) is the author/funder, who has granted bioRxiv a license to display the preprint in perpetuity. It is made available under aCC-BY 4.0 International license.

Suppl Figure 3

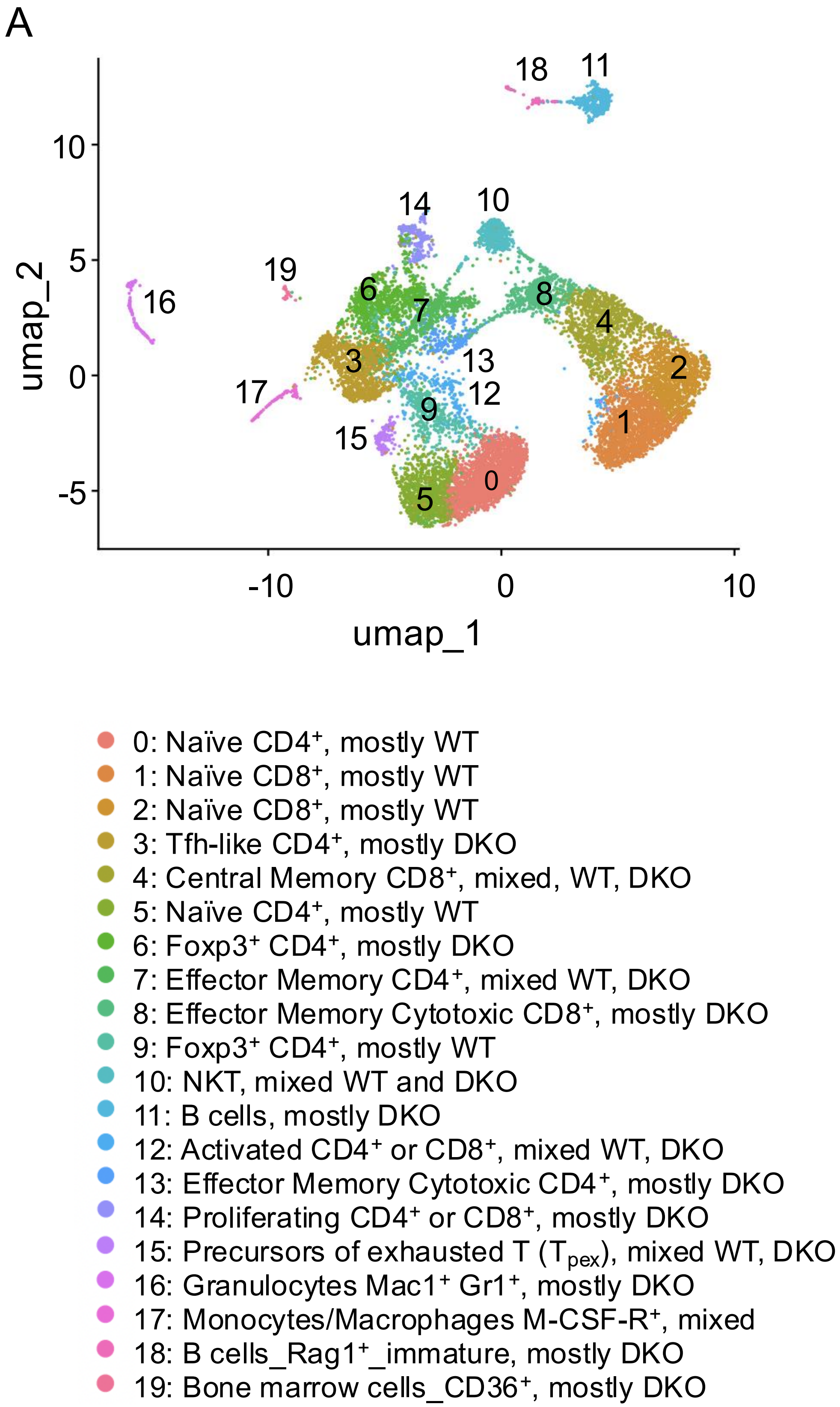

bioRxiv preprint doi: https://doi.org/10.1101/2025.08.29.673187; this version posted September 3, 2025. The copyright holder for this preprint (which was not certified by peer review) is the author/funder, who has granted bioRxiv a license to display the preprint in perpetuity. It is made available under aCC-BY 4.0 International license.

Suppl Figure 4

A

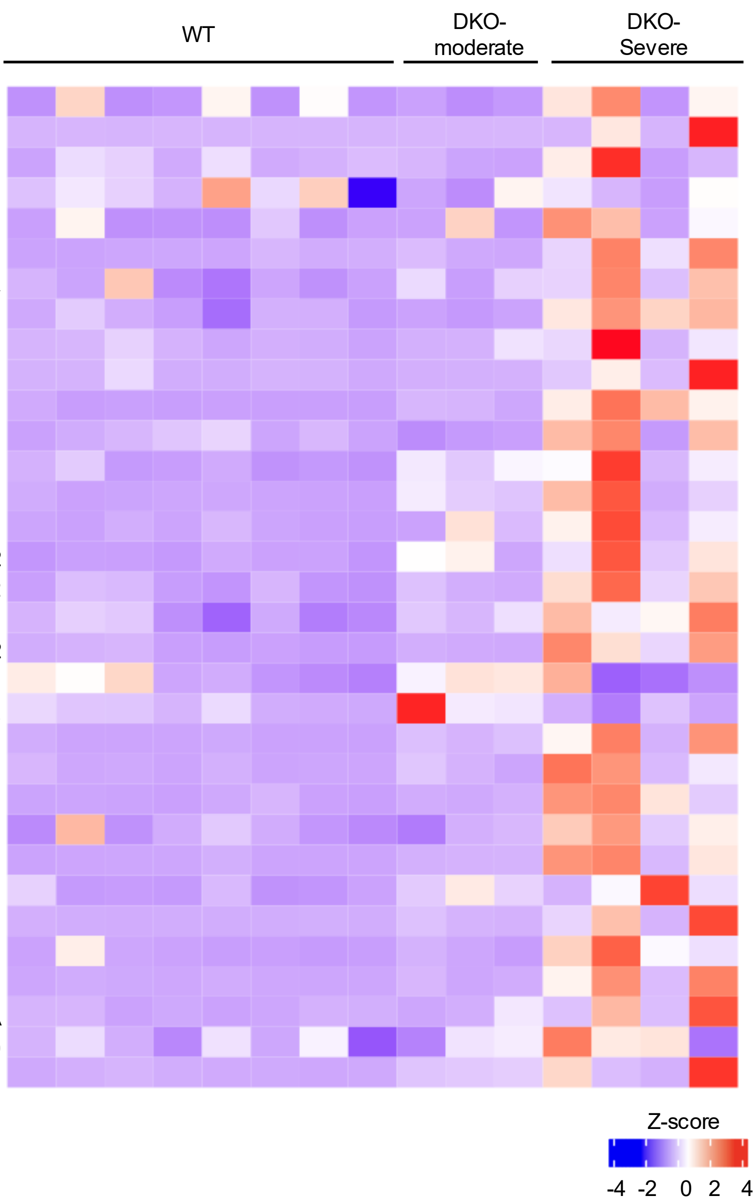

	Significance		
	WT vs DKO-M	WT vs DKO-S	DKO-M vs DKO-S
Alanyl-tRNA Synthetase (PL-12)	NO	NO	NO
CCP peptide	NO	YES	NO
Centromere B (CENP-B)	NO	NO	NO
Centromere Protein A (CENP-A)	NO	NO	NO
Gliadin	NO	YES	NO
Glycyl-tRNA Synthetase (EJ)	NO	YES	YES
Histone H2A	NO	YES	NO
Histone H2B	NO	YES	YES
Jo-1 (histidyl tRNA synthetase)	NO	YES	NO
KRT19	NO	YES	NO
KRT20	NO	YES	YES
KRT8	NO	YES	YES
La/SS-B	NO	YES	NO
Liver cytosolic antigen type 1 (LC-1)	NO	YES	NO
M/Scl 100	NO	YES	NO
M2	NO	YES	NO
Mi-2	NO	YES	YES
Myeloperoxidase (MPO), p-ANCA	NO	YES	YES
Nup62	NO	YES	YES
Proliferating Cell Nuclear Antigen (PCNA)	NO	NO	NO
Proteinase-3 (PR3), c-ANCA	NO	NO	NO
Ro/SS-A (52 kDa)	NO	YES	YES
Ro/SS-A (60 kDa)	NO	YES	YES
Scl-70 (DNA topoisomerase-1)	NO	YES	YES
Sm/Smith/snRNP core protein	NO	YES	YES
Threonyl-tRNA synthetase (PL-7)	NO	YES	YES
Thyroglobulin (TG)	NO	YES	NO
Thyroid peroxidase (TPO)	NO	YES	NO
Tissue Transglutaminase (tTG)	NO	YES	YES
U1-snRNP70 kDa	NO	YES	YES
dsDNA	NO	YES	NO
gp-210	NO	NO	NO
sp100	NO	YES	NO

bioRxiv preprint doi: https://doi.org/10.1101/2025.08.29.673187; this version posted September 3, 2025. The copyright holder for this preprint (which was not certified by peer review) is the author/funder, who has granted bioRxiv a license to display the preprint in perpetuity. It is made available under aCC-BY 4.0 International license.

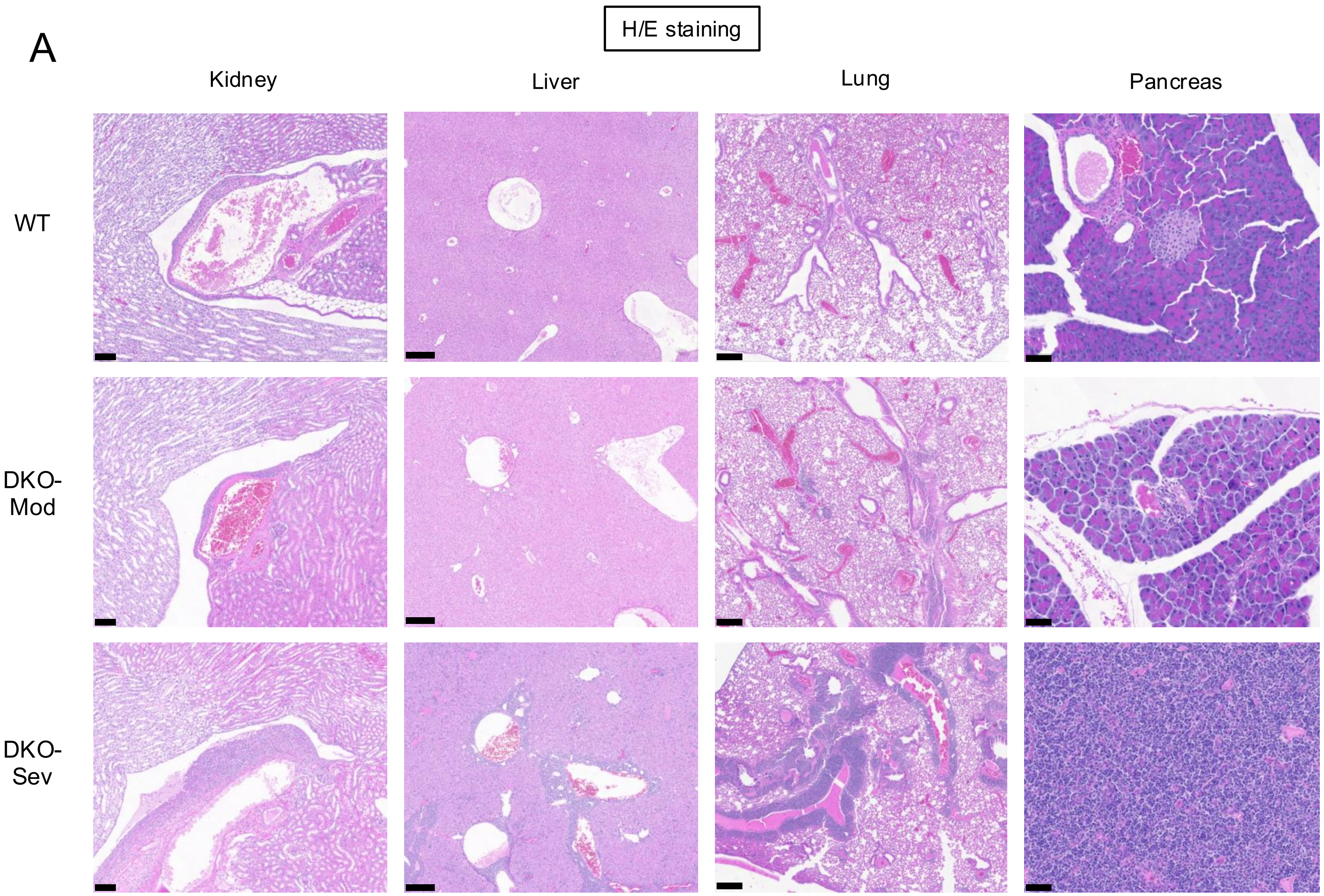

bioRxiv preprint doi: https://doi.org/10.1101/2025.08.29.673187; this version posted September 3, 2025. The copyright holder for this preprint (which was not certified by peer review) is the author/funder, who has granted bioRxiv a license to display the preprint in perpetuity. It is made available under aCC-BY 4.0 International license.

Suppl Figure 6

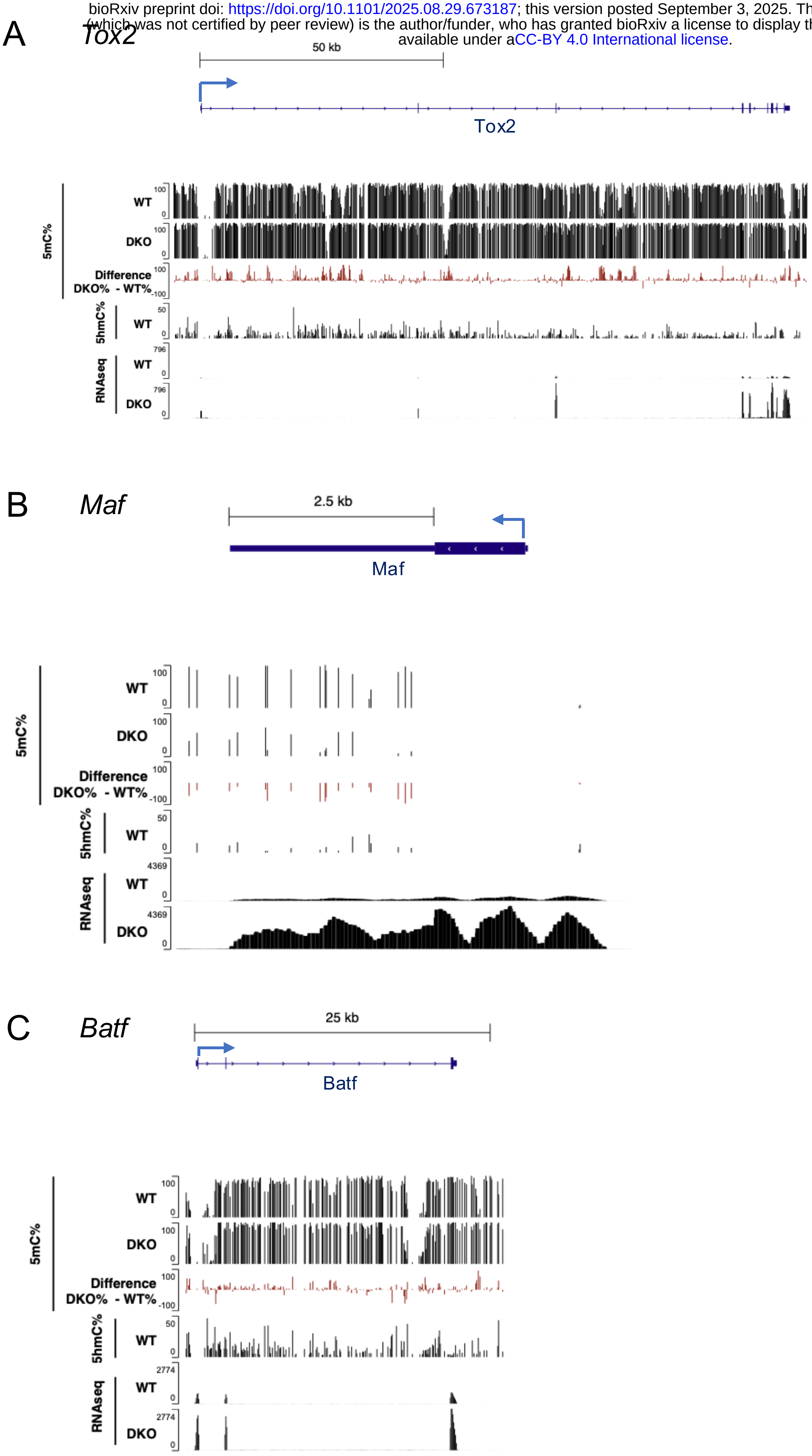

Supplement: Supplement 1 [file NIHPP2025.08.29.673187v1-supplement-1.pdf]
